# De Winter Sign as an Anterior ST-Segment Elevation Myocardial Infarction Equivalent

**DOI:** 10.1016/j.jaccas.2026.107360

**Published:** 2026-05-20

**Authors:** David T. Zhang, Chad Gier, Mark Hellerman

**Affiliations:** a Division of Cardiology, Department of Medicine, Stony Brook Medicine, Stony Brook, New York, USA; b Division of Cardiology, Department of Medicine, Northwestern Medicine, Chicago, Illinois, USA

**Keywords:** de Winter sign, STEMI

## Abstract

**Introduction:**

de Winter sign is a well-recognized electrocardiographic pattern that represents an acute proximal left anterior descending (LAD) coronary artery occlusion. Despite the absence of a classic ST-segment elevation, it is considered an ST-segment elevation myocardial infarction equivalent and requires emergent reperfusion therapy.

**Case Presentation:**

We report the case of an older patient presenting with acute chest pain whose initial electrocardiogram demonstrated de Winter sign. Prompt recognition led to emergent coronary angiography, confirming proximal LAD occlusion, and successful percutaneous coronary intervention. His course was complicated by cardiogenic shock requiring mechanical circulatory support.

**Discussion:**

This case highlights the importance of early recognition of the de Winter sign, representing acute proximal LAD occlusion despite the absence of an ST-segment elevation, to avoid delays in reperfusion therapy and improve clinical outcomes.

## Graphical Abstract

**Figure:**
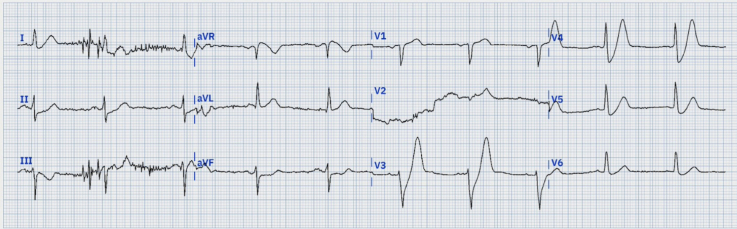

## History of Presentation

A 70-year-old man with a mood disorder, remote prostate cancer, and remote cutaneous T-cell lymphoma presented with sudden-onset chest pain radiating to his jaw for 1 hour. Presenting vital signs were notable for a heart rate of 66 beats per minute, blood pressure 94/50 mm Hg, respiratory rate 22 breaths per minute, and normal peripheral oxygen saturation and temperature. Physical examination was notable for a diaphoretic, unwell-appearing man.

**Take-Home Messages**

• de Winter sign represents acute proximal left anterior descending occlusion despite the absence of ST-segment elevation.
• de Winter sign is a “STEMI equivalent” and should prompt emergent reperfusion therapy.

**Figure 1:**
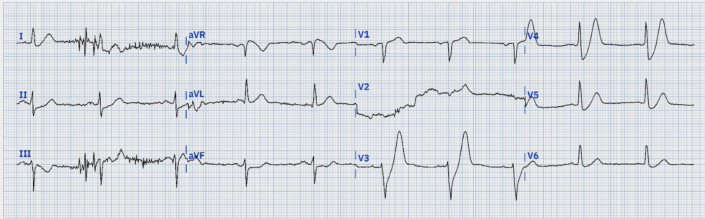
Presenting Electrocardiogram With de Winter Sign

An initial electrocardiogram (ECG) was obtained (Figure 1). Current medications included lamotrigine, bupropion, fluvoxamine, and alprazolam. Laboratory values revealed normal initial troponin, electrolytes, and blood counts.

## Question

Which of the following is the most accurate interpretation of this ECG finding?

A. Non-ST-segment elevation myocardial infarction (NSTEMI)
B. de Winter sign (STEMI equivalent)
C. Early repolarization pattern
D. Posterior myocardial infarction

## Discussion and Rationale

Prompt recognition of the de Winter sign as a “STEMI equivalent” led to emergent coronary angiogram—confirming single-vessel disease with 100% occlusion of the proximal left anterior descending (LAD)—requiring a drug-eluting stent, dual antiplatelet therapy with aspirin and ticagrelor, and lipid-lowering therapy with high-dose atorvastatin and ezetimibe.

Choice A is incorrect. While an NSTEMI can show ST-segment depressions or T-wave changes, the de Winter sign specifically represents complete LAD occlusion. Mislabeling this ECG as an NSTEMI could delay emergent reperfusion.

Choice B is correct. The de Winter sign, first described in 2008, is an ECG pattern strongly associated with acute proximal LAD occlusion.^1^ Failure to recognize this entity may result in delayed treatment and increased myocardial injury. Key ECG features include upsloping ST depression at the J point in the precordial leads, prominent and symmetric hyperacute T waves, absence of contiguous ST-segment elevations, and possible ST-segment elevation in lead aVR.^1^^,^^2^ Our patient's ECG demonstrates all of these features.

Choice C is incorrect. Early repolarization is usually benign and appears as concave ST-segment elevations in the precordial leads with J-point notching, lacking the characteristic hyperacute T waves and J-point depression of a de Winter sign.

Choice D is incorrect. Posterior myocardial infarctions typically demonstrate horizontal or downsloping ST depressions that are maximal in leads V_1_ to V_4_ compared to those in V_5_ and V_6_. This pattern contrasts with the upsloping ST-segment depression with hyperacute T waves seen in the de Winter sign.

Beyond its diagnostic and therapeutic importance, the de Winter sign carries significant implications for systems-based care and clinical decision-making. Because current STEMI activation criteria in many institutions rely heavily on the presence of ST-segment elevation, patients with de Winter signs are at risk of delayed catheterization and worse outcomes if the ECG is misclassified as non–ST-segment elevation acute coronary syndrome.^3^ Increasing awareness through targeted ECG education, protocol updates recognizing STEMI equivalents, and early cardiology consultation may reduce treatment delays and improve myocardial salvage in this high-risk population.

This case underscores the de Winter sign as a STEMI equivalent requiring urgent recognition and management. Emergency physicians and cardiologists should maintain a high index of suspicion when encountering this ECG pattern in patients with ischemic symptoms to prevent delays in life-saving reperfusion therapy.

## Funding Support and Author Disclosures

The authors have reported that they have no relationships relevant to the contents of this paper to disclose.
